# Prognosis of the intrahepatic cholangiocarcinoma after resection: hepatitis B virus infection and adjuvant chemotherapy are favorable prognosis factors

**DOI:** 10.1186/1475-2867-13-99

**Published:** 2013-10-18

**Authors:** Rui-qing Liu, Shu-jing Shen, Xiu-feng Hu, Jie Liu, Li-juan Chen, Xing-ya Li

**Affiliations:** 1 First Affiliated Hospital of Zhengzhou University, Zhengzhou 450052, China; 2 He Nan Provincial people's Hospital/Affiliated People's Hospital of Zhengzhou University, Zhengzhou 450003, China; 3 He Nan Province Tumor hospital/Affiliated Tumor Hospital of Zhengzhou University, Zhengzhou 450008, China

**Keywords:** Intrahepatic cholangiocarcinoma, Hepatitis B virus, Adjuvant chemotherapy, Survival, Prognosis

## Abstract

**Aim:**

The incidence and mortality associated with intrahepatic cholangiocarcinoma is increasing in many countries and documentation of disease outcome is sparse. The present study was undertaken to investigate the prognostic factors for intrahepatic cholangiocarcinoma (ICC) following surgical resection. The impact of pre-existing HBV virus infection and adjuvant chemotherapy on the overall survival was also evaluated.

**Methods:**

Clinical and pathological data were collected retrospectively from 81 patients undergoing surgery for ICC between 2005 and 2011, at The Henan Province Tumor Hospital and the First Affiliated Hospital of Zheng Zhou University. Survival and prognosis were analyzed using the Kaplan-Meier method and COX regression model.

**Results:**

The population included 37 patients who were HBsAg + or anti-HBc+, 21 patients who were anti-HBs + positive and 18 patients who received adjuvant chemotherapy. The overall 1- and 3-year survival rates were 51% and 20%, respectively. The median survival was 12.2 months. Univariate analysis identified the following prognostic factors: HBV virus infection or HBV vaccine prior to resection (P = 0.017); adjuvant chemotherapy (P = 0.001); preoperative serum CA19-9 (> 200 U/mL; P = 0.015); GGT (> 64 U/L; P = 0.008), ALP (> 119 U/L; P = 0.01); lymph node metastasis (P = 0.005); radical resection (P = 0.021); intrahepatic metastasis (P = 0.015) and diabetes (P = 0.07). Multivariate analysis identified chronic HBV infection (RR = 0.583; P = 0.041), anti-HBs positivity (RR = 0.680; P = 0.050), adjuvant chemotherapy (RR = 0.227; P < 0.001), lymph node metastasis (RR = 2.320; P = 0.001), and intrahepatic duct stones (RR = 0.473; P = 0.032) as independent prognostic factors.

**Conclusions:**

HBV virus infection or HBV vaccination prior to resection, together with adjuvant chemotherapy, were independently associated with improved survival in patients undergoing surgery for ICC.

## Introduction

Intrahepatic cholangiocarcinoma (ICC) is the second most common form of primary hepatic tumor accounting for 3.3% of all such cancers. It originates from epithelial cells located in the intrahepatic bile duct or at the end of the bile duct. The incidence and mortality associated with ICC are increasing in many countries [1]. Due to the lack of typical symptoms that define early stage disease, many patients present with lymph node metastasis at the time of diagnosis. These patients are not suitable candidates for hepatic resection which is the only therapy associated with prolonged survival [2]. Consequently prognosis is generally poor.

Recent studies have identified HBV infection as an independent risk factor for ICC [3,4]. However the impact of HBV infection on the outcome ICC remains unclear [5,6]. In the present study we investigated the impact of HBV infection on the survival of patients undergoing surgical resection for ICC. Generally speaking, anti-hepatitis B core (HBc)-positivity alone is either indicative of previous HBV infection or the disappearance of hepatitis B surface antigen (HBsAg). Anti-HBs, therefore, provides evidence of recent infection, and acts as a sentinel marker for HBV infection (HBsAg carrier). Occult HBV infection is sometimes identified by molecular techniques even in patients who show HBsAg seronegativity [7,8]. However, previous hepatitis B vaccination does not result in anti-HBc, because the components of hepatitis B vaccine do not relate to hepatitis B core antigen (HBcAg). Thus, the consensus is that anti-HBc + alone indicates current or previous HBV infection, while anti-HBs positivity is indicative of previous infection or injection of HBV vaccine.

Much controversy surrounds the use of adjuvant chemotherapy for ICC. Most data come from small, uncontrolled trials that include cases of gallbladder, pancreatic and biliary system carcinoma [9], making it difficult to draw meaningful conclusions regarding efficacy. In addition, there is no consensus about standard chemotherapy.

## Materials and methods

### Patients

Clinical and pathological data were retrospectively collected from 81 patients who underwent surgical resection of pathologically confirmed ICC between January 2005 and December 2011 at the Henan Province Tumor Hospital and the First Affiliated Hospital of Zheng Zhou University. Patients who had received pre-operative chemotherapy and those with hilar choangiocarcinoma or HCV infection were excluded from the analysis.

The population included 48 men and 33 women, with a median age of 59 years (range: 30 to 76 years). Fourteen patients (17.2%) were heavy drinkers, 27 patients (33.3%) were smokers and six (7.4%) had diabetes.

In total, 37 patients (45.7%) were anti-HBc + or HBsAg+, 21 (25.9%) were anti-HBs + and 23 patients (28.4%) were negative for all five makers. Among the 37 anti-HBc + patients eight were also Anti-HBs positive. A significant proportion of patients presented with abnormal preoperative levels of liver function markers. Thirty six (44.4%) patients had elevated ALT; AST was elevated in 37 patients (45.7%), alkaline phosphatase in 51 patients (63.0%), GGT in 58 patients (71.6%), total bilirubin in 29 patients (35.8%) and albumin in 23 patients (28.4%). In terms of tumor markers, 57 patients (70%) had CA19-9 levels above 37 U/mL, 47 (54.3%) had CA19-9 levels above 200 U/mL and eight patients (6.7%) had elevated alpha fetoprotein levels.

Imaging studies and surgical records showed that 50 patients (61.7%) had regional lymph node metastases; 31 (38.3%) had intrahepatic metastases, and 13 (16.0%) had intrahepatic duct stones. Eighteen patients received adjuvant chemotherapy after surgery. Twelve 12 patients received the transhepatic arterial chemotherapy and embolization (TACE), and six received intravenous chemotherapy.

### Statistical analysis

All the patients were followed up by the telephone or mail until death or the study cut-off (20 October 2012).

Statistical analysis was performed using the SPSS version 17.0 software. Overall survival time was calculated from the date of surgery using the Kaplan-Meier method. Survival rates between groups were compared using log-rank and multivariate regression analysis. The Cox proportional hazards model was used to identify independent prognostic factors. Values of P < 0.05 were considered statistically significant.

## Results

### Overall survival

Patient survival ranged from 1 to 62 months (median: 12.2 months) with five patients still alive at the end of the follow-up period. The overall 1- and 3-year survival rates were 51% and 20%, respectively. The median survival time was 12.2 months.

### The impact of HBV infection and vaccine on postoperative outcome

**Figure 1:**
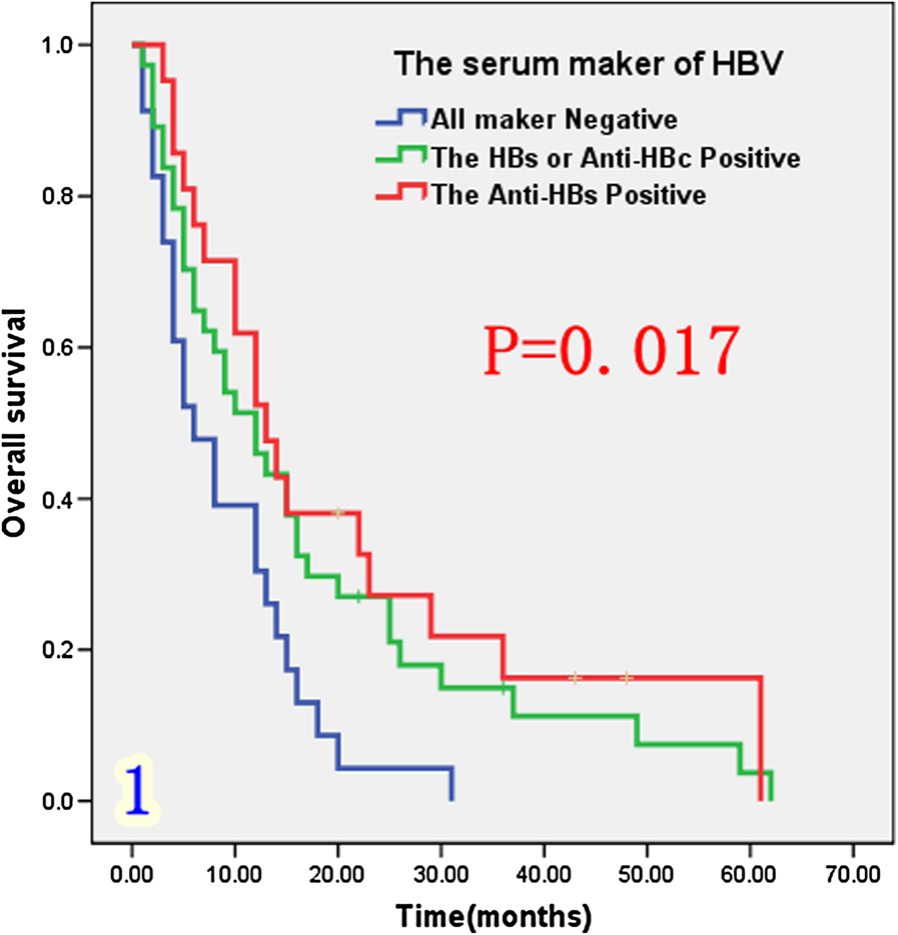
Overall survival rates after the surgical resection in 81 ICC patients, 3 groups according to the 5 markers of HBV in serum.

The population was divided into three groups according to the serum HBV markers. The first group who were HBs-Ag or anti-HBc positive, represented patients with previous or current infection; the second group who were anti-HBs positive only, represented those with HBV infection or previous vaccination and the third group comprised patients with negative serum markers of HBV, who had never been infected with HBV. As shown in Figure 1 and Table 1, median survival was longer in patients who were anti-HBc and/or anti HBs positive (12 to 13 months) than in those with negative markers (6 months; P = 0.017). Survival among the eight HBsAb positive patients was similar to that among HBs-Ag or anti-HBc positive patients.

**Table 1:** Univariate analysis of overall survival following resection for ICC (n = 81)

**Clinical factor**	**N**	**Survival rate (%)**		**Median survival (months)**	**P-value (log-rank)**
		**1-year**	**3-year**		
***Gender***					0.608
Male	48	39.6%	9.2%	10	
Female	33	48.5%	13.9%	12	
***Age***					0.108
≤ 60 years	43	48.8%	15.2%	12	
> 60 years	38	36.8%	6.3%	7	
***Intrahepatic duct stones***					0.277
Yes	13	53.8%	15.4%	26	
No	68	41.5%	10.7%	10	
***Serum hepatitis index***					0.017
HBsAg or anti-HBc	37	45.9%	15.0%	12	
anti-HBs +	21	52.4%	16.3%	13	
All -	23	30.4%	0%	6	
***ALT***					0.068
≤ 40 U/L	45	53.3%	13.2%	13	
> 40 U/L	36	31.6%	9.8%	10	
***CA19-9***					0.015
≤ 200 U/ml	34	50.0%	19.1%	12	
> 200 U/ml	47	38.3%	5.1%	8	
***GGT***					0.008
≤ 64 U/L	23	60.9%	19.1%	16	
> 64 U/L	58	36.2%	7.9%	9	
***Total bilirubin***					0.124
≤ 20 μmol/L	52	48.0%	13.6%	12	
> 20 μmol/L	29	34.5%	6.9%	9	
***Alkaline phosphatase***					0.01
≤ 119 U/L	30	60.0%	16.2%	15	
> 119 U/L	51	33.3%	7.8%	8	
***Lymphatic metastasis***					0.005
Yes	50	34.0%	6.0%	6	
No	31	58.1%	20.2%	15	
***Operative procedure***					0.021
Radical	60	45.0%	14.2%	12	
Palliative	21	28.6%	0	5	
***Intrahepatic metastasis***					0.015
Yes	31	29.0%	6.5%	6	
No	50	52.0%	13.9%	13	
***Diabetes***					0.07
Yes	6	16.7%	0	5	
No	75	45.3%	12.0%	12	
***Adjuvant chemotherapy***					0.001
Yes	18	83.8%	33.3%	22	
No	63	31.7%	4.1%	8	

### The impact of adjuvant chemotherapy on the ICC patients

**Figure 2:**
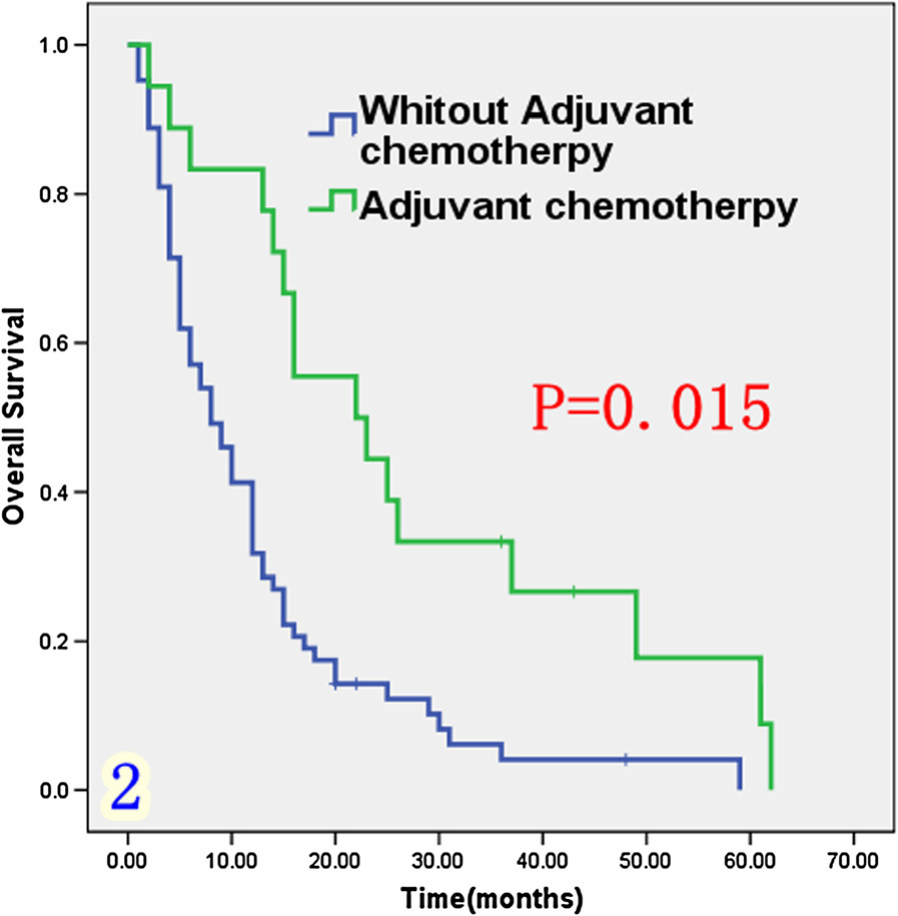
Overall survival rate in 81 ICC patients according to received adjuvant chemotherapy or not.

Eighteen patients received adjuvant chemotherapy (Table 1) including 12 patients who underwent transcatheter hepatic arterial chemoembolization (TACE) and six patients who received systemic venous antineoplastic therapy. Chemotherapy included 5-FU, cisplatin, gemcitabine, doxorubicine and oxaliplatin. As shown in Figure 2, median survival was significantly longer among patients receiving adjuvant chemotherapy (22 months) than among those not receiving chemotherapy (8 months; P = 0.01).

### Effect of prognostic factors on survival

**Figure 3:**
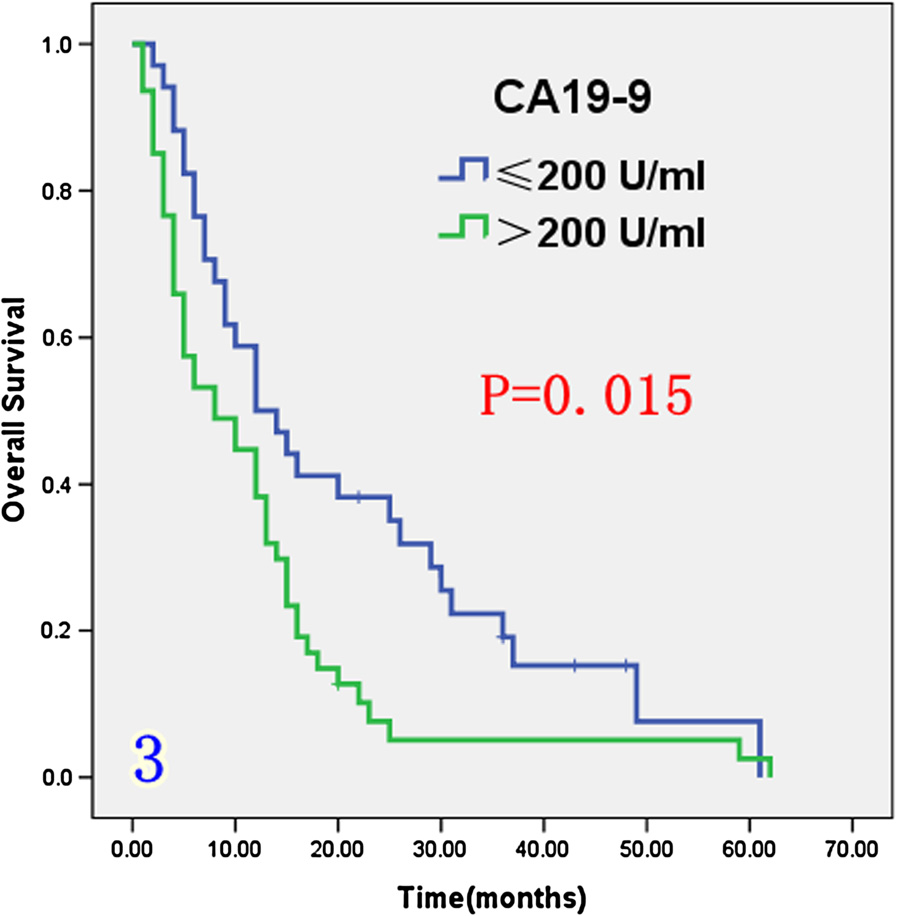
Overall survival rate of in 81 ICC patients after surgery according to the serum level of CA19-9 from the Kaplan-Meier method.

**Figure 4:**
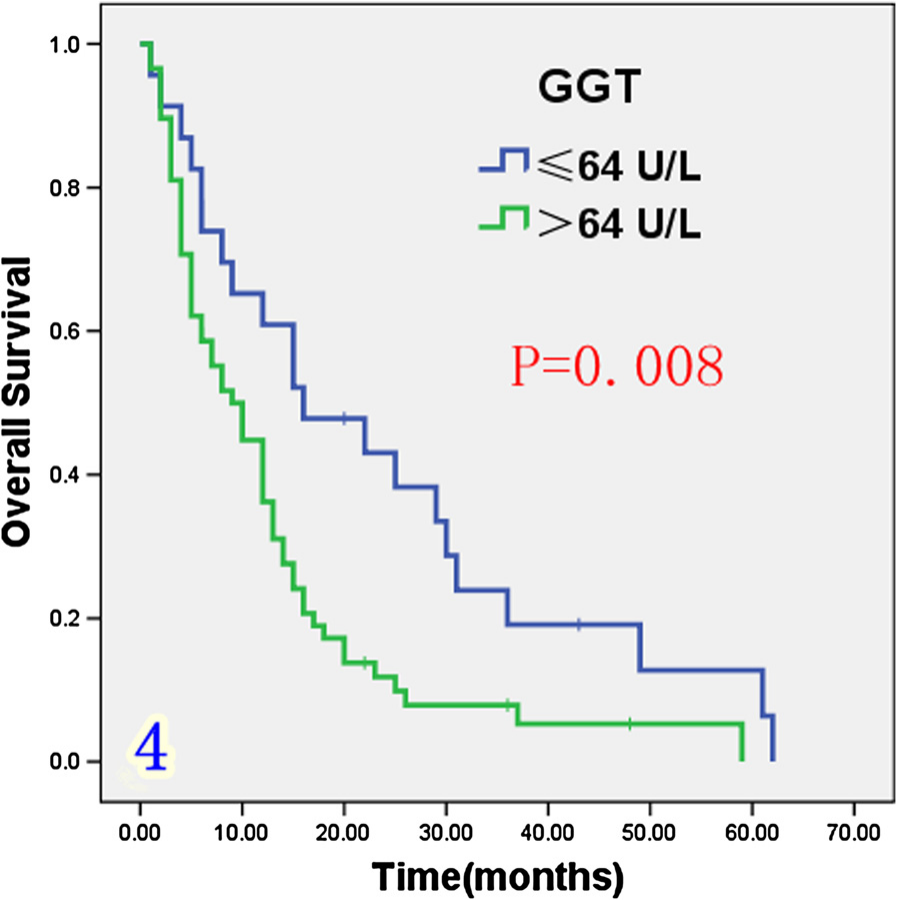
Overall survival rate of in 81 ICC patients after surgery according to the serum level of GGT from the Kaplan-Meier method.

**Figure 5:**
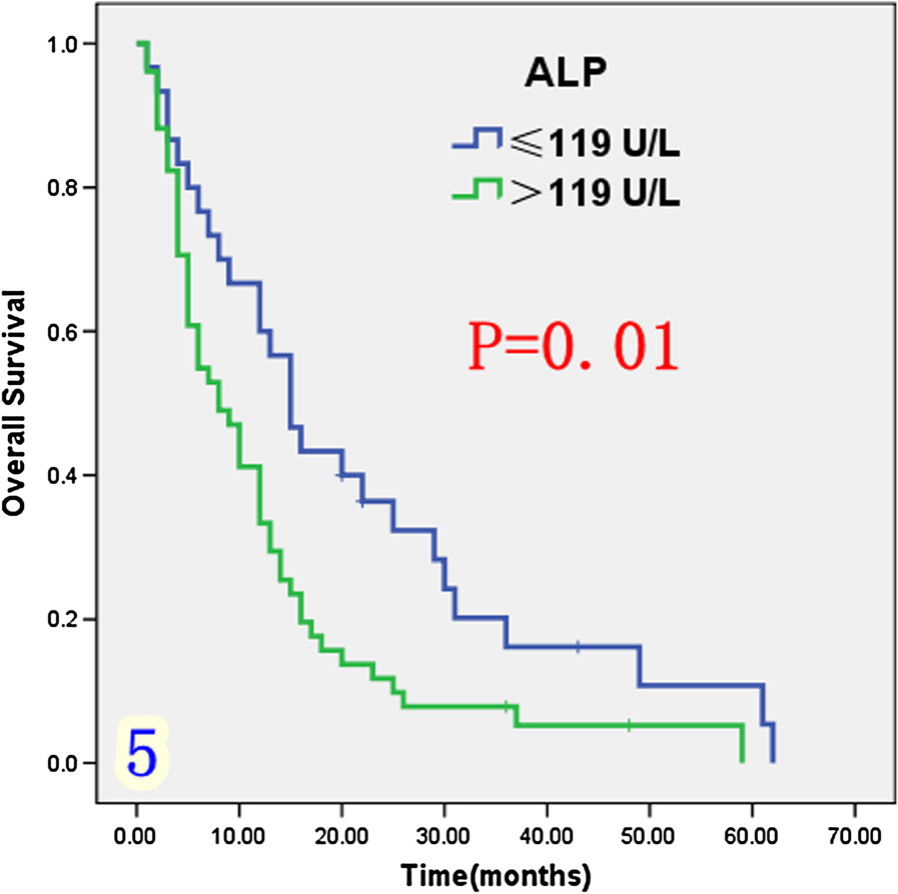
Overall survival rate of in 81 ICC patients after surgery according to the serum level of ALP from the Kaplan-Meier method.

**Figure 6:**
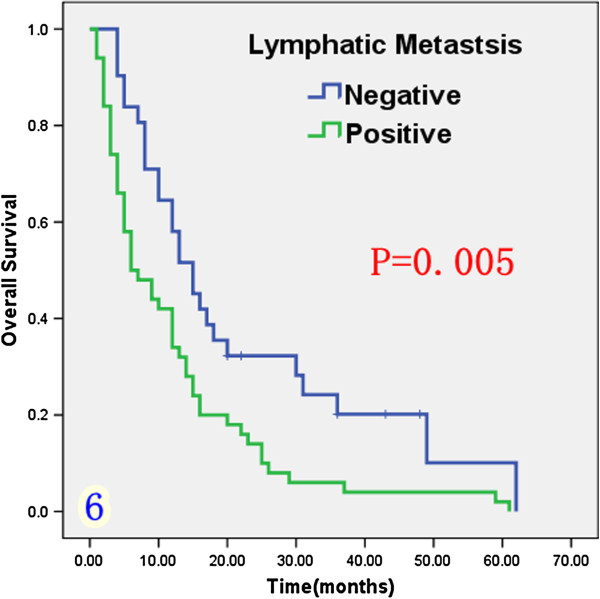
Overall survival rate of in 81 ICC patients after surgery according to the lymph node metastasis or not from the Kaplan-Meier method.

**Figure 7:**
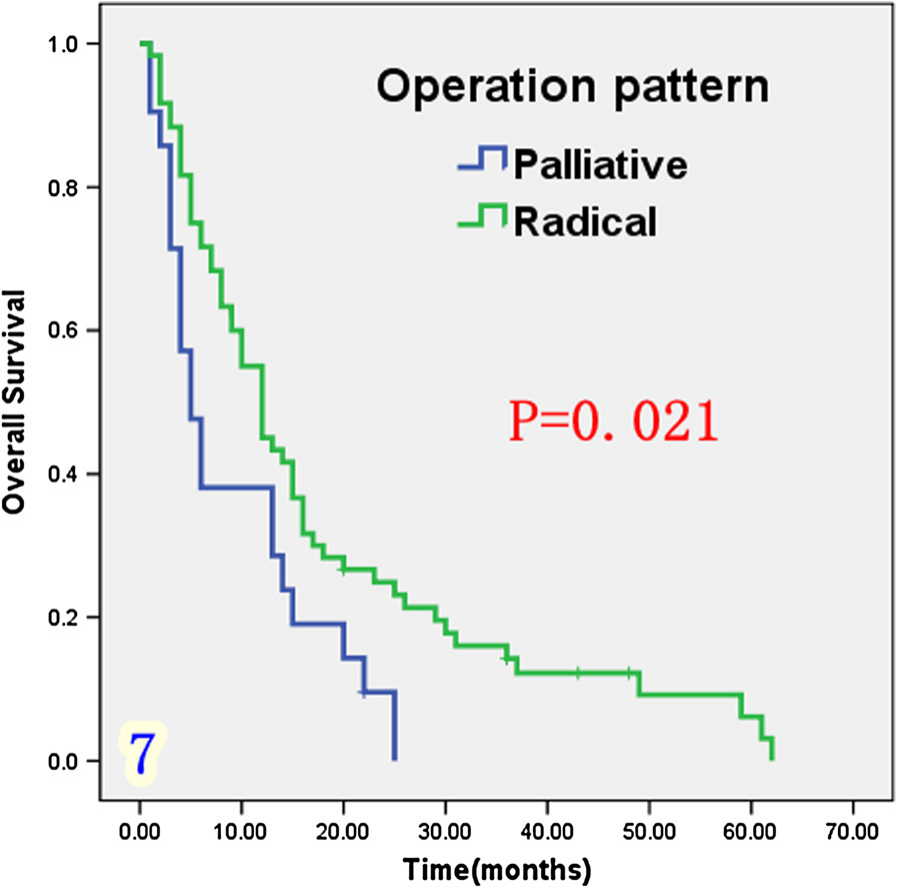
Overall survival rate of in 81 ICC patients after surgery according to the operation pattern from the Kaplan-Meier method.

**Figure 8:**
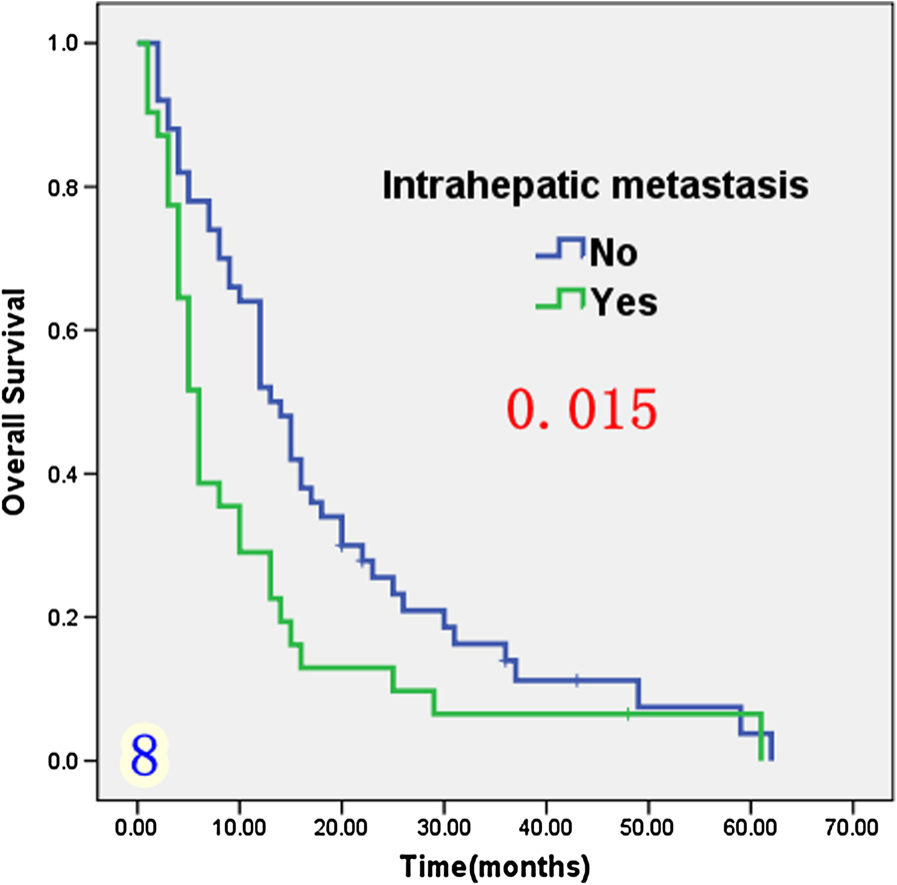
Overall survival rate of in 81 ICC patients after surgery according to the intrahepatic metastasis or not from the Kaplan-Meier method.

**Figure 9:**
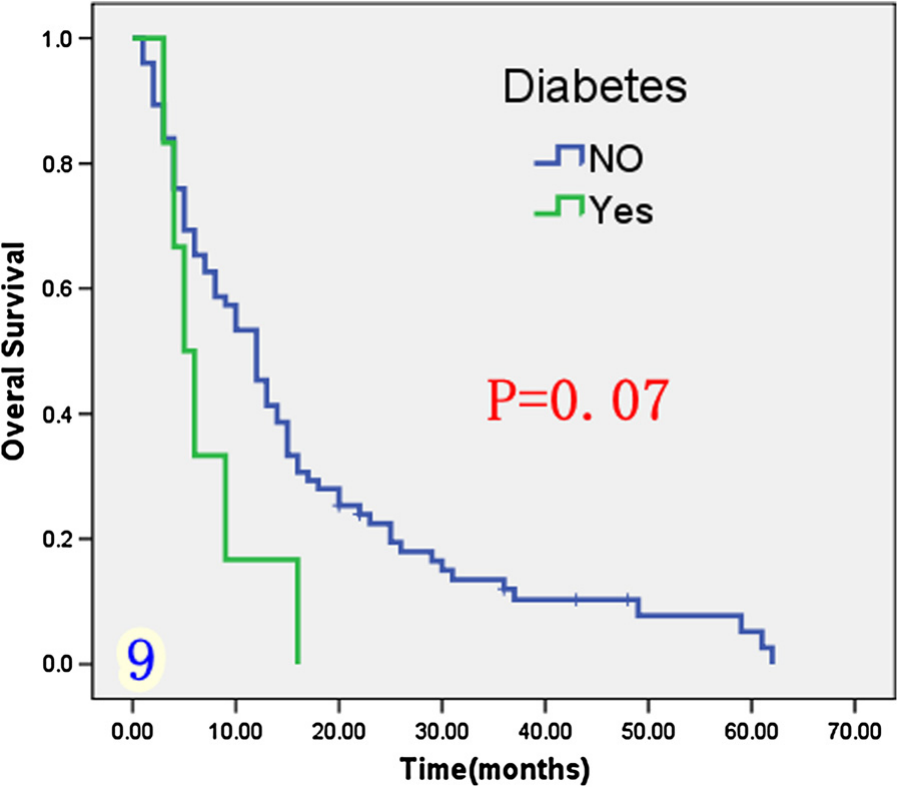
Overall survival rate of in 81 ICC patients after surgery according to with diabetes or not from the Kaplan-Meier method.

Univariate analysis (Table 1) indicated that preoperative serum levels of CA19-9 (≥ 200 U/L), GGT (≥ 64 U/L) and ALP (≥ 119 U/L) were associated with improved survival (Figures 3, 4 and 5). Other factors linked to survival benefit were lymph node metastasis, radical surgery, intrahepatic metastasis and diabetes (Figures 6, 7, 8 and 9).

Multivariate analysis (Table 2) identified chronic HBV infection (RR = 0.583; P = 0.041), anti-HBs positivity (RR = 0.680; P = 0.050), adjuvant chemotherapy (RR = 0.227; P < 0.001) and intrahepatic duct stones (RR = 0.473; P = 0.032) as favorable independent prognostic factors while the presence of lymph node metastases was associated with poor overall survival (RR = 2.320; P = 0.001).

**Table 2:** Multivariate analysis of overall survival following resection for ICC (n = 81)

**Variate**	**B**	**SE**	**Wald**	**df**	**P**	**Exp(B)**	**95%CI**
***Chronic HBV infection***	−0.539	0.263	4.193	1	0.041	0.583	0.384-0.977
***The anti-HBs positive***	−0.680	0.274	3,256	1	0.050	0.650	0.423-0.994
***Lymphatic metastasis***	0.841	0.262	10.276	1	0.001	2.320	1.387-3.880
IHDS^*^	−0.749	0.349	4.597	1	0.032	0.473	0.239-0.938
***Adjuvant chemotherapy***	−1.481	0.337	19.294	1	0.000	0.227	0.117-0.440

*IHDS intrahepatic duct stone, *TBIL* Total bilirubin, *ALT* Alanine aminotransferase, *ALP* Alkaline phosphatase, *GGT* gamma-glutamyl transpeptidase, *CA19-9* Carbohydrate antigen 19–9.

### Stratification of prognostic factors by HBV status

Based on the preceding results we analyzed differences in the distribution of each of the prognostic factors according to the serum HBV status. Abnormal preoperative serum CA19-9 (≥ 200 U/L) was present in 43.4% patients with chronic HBV and in 69.6% patients without HBV infection (P = 0.047). Elevated ALP levels were present in 29.7% of patients with HBV infection and in 87% of patients without HBV infection (P = 0.001), and lymph node metastases were present in 73.2% of patients without HBV infection and in 46.0% of those with chronic HBV infection (P = 0.001).

The differences in the distribution of these factors in patients without HBV infection and in those who were anti-HBs positive did not reach statistical significance although numerical differences were apparent (Table 3).

**Table 3:** Stratification of prognostic factors by HBV status

**Clinical factor**	**Chronic hepatitis**	**P value**	**All makers negative**	**P value**	**The anti-HBs positive**
***Total***	37		23		21
***Age***		0.051		0.690	
≤ 60 y	24 (67.2%)		9 (39.1%)		7 (29.2%)
> 60 y	13 (32.8%)		14 (60.9%)		14 (70.8%)
***CA19-9***		0.047		0.622	
≤ 200 U/ml	21 (56.8%)		7 (30.4%)		5 (23.8%)
> 200 U/ml	16 (43.2%)		16 (69.6%)		16 (76.2%)
***TIBL***		0.051		0.131	
≤ 20 μmol/L	24 (64.9%)		9 (39.1%)		13 (61.9%)
> 20 μmol/L	13 (35.1%)		14 (60.9%)		8 (38.1%)
***GGT***		0.496		0.870	
≤ 64 U/L	11 (29.7%)		5 (21.7%)		5 (23.8%)
> 64 U/L	26 (70.3%)		18 (78.3%)		16 (76.2%)
***ALP***		0.001		0.202	
≤ 119 U/L	16 (43.2%)		3 (13.0%)		6 (28.6%)
> 119 U/L	11 (56.8%)		20 (87.0%)		15 (71.4%)
***Lymphatic metastasis***		0.001		0.862	
No	20 (54.0%)		6 (26.8%)		5 (23.8%)
Yes	17 (46.0%)		17 (73.2%)		16 (76.2%)
***Operation pattern***		0.518		0.717	
Radical	24 (64.9%)		13 (56.5%)		13 (61.9%)
Palliative	13 (35.1%)		10 (43.5%)		8 (38.1%)
***Intrahepatic metastasis***		0.811		0.820	
No	23 (62.1%)		15 (65.2%)		13 (61.9%)
Yes	14 (37.9%)		8 (34.8%)		8 (38.1%)
***Adjuvant chemotherapy***	8 (21.6%)		5 (21.7%)		5 (23.8%)

## Discussion

The prognosis of ICC remains poor and surgery offers the only chance of a clinical cure [2]. Although many traials have reported that cytokine-induced killer (CIK) cell therapy is safety and effective for hepatocellular carcinoma [10], its use in ICC requires further study.

The overall 1- and 3-year survival rate for our series of 81 patients (51% and 20%, respectively) was lower than that reported by other recent studies [5,6,11]. The reasons for poor survival in our study were related to the fact that approximately 26.0% of patients were not well enough to undergo radical surgery. In addition, 61.7% presented with lymphatic metastasis, 38.3% had intrahepatic metastasis and between 35.8% and 71.6% patients had abnormal liver function at the time of diagnosis.

We showed that HBV infection or vaccine prior to surgery were favorable prognostic factors for survival after resection. Patients with occult HBV infection represented by HBsAg + or anti-HBc + (n = 37) and those who had undergone vaccination (n = 21) had a better prognosis than patients without HBV infection or injection prior to surgery (n = 23). Multivariate analysis indicated that chronic HBV infection and anti-HBs positivity both acted as favorable prognostic factors for overall survival.

Previous studies have investigated the association between chronic HBV infection and ICC [3,4]. However, the true impact of HBV on survival of patients with ICC remains unclear [5,6]. It has been proposed that some of the mechanisms which underlie ICC related to HBV infection might be similar to those responsible for hepatocellular cancer (HCC) which accounts for the shared clinical features of ICC and HCC [12]. It has been speculated that ICC might originate from a hepatic precursor cell with a hidden multi-differentiation potential, that can be activated to proliferate and differentiate into mature hepatocytes or biliary cells [13]. It is also possible that fragments of HBV gene may integrate into the host genome, resulting in cellular transformation and recruitment of hepatocytes or cholangiocytes with oncogenic potential. Another possibility is that innate or acquired immune responses activated by current or recent HBV infection, might enhance antitumor activity against ICC. ICC associated with HBV infection, may activate immunologic memory arising from previous HBV infection and thereby strengthen antitumor immunity [14].

Vaccination is a protective strategy against HBV infection and the resulting immune responses may explain why prognosis was improved in patients who had been vaccinated. However, the exact mechanisms involved require further study.

The prognosis of ICC was associated with the presence or absence of HBV infection suggesting that the pathogenesis and tumor microenvironment were different in these subgroups of patients. Based on this finding, patients with HBV infection may be sensitive to antiviral or immunological therapy, which may contribute to the control of the disease.

The use of adjuvant chemotherapy and radiation in ICC remains controversial [15,16]. Most previous studies of adjuvant chemotherapy in ICC are uncontrolled, with small sample sizes and include patients with gallbladder and pancreatic cancer making it difficult to draw clinically meaningful conclusion regarding efficacy. In previous studies, 5-FU based chemotherapy resulted in response rates of 8%-40%, and a median survival of 2 to 12 months. Gemcitabine-based chemotherapy is associated with response rates ranging from 8% to 60% and median survival times ranging from 6.5 to 16 months [9]. In present study, multivariate analysis identified adjuvant chemotherapy was an independent prognostic factor which was associated with an improved median survival of 15 months. This finding suggests that high risk patients with ICC should receive adjuvant chemotherapy.

In our study 18 patients received adjuvant chemotherapy including 12 who underwent transcatheter hepatic arterial chemoembolization (TACE) and six patients who received systemic venous antineoplastic therapy. Chemotherapy included 5-FU, cisplatin, gemcitabine, doxorubicine and oxaliplatin. The impact of individual chemotherapy regimens on prognosis and survival in patients with ICC requires further study in larger populations of patients.

High preoperative CA19-9 levels (> 37 U/ml) have been shown to influence overall survival of ICC patients after hepatic resection [17]. Serum CA19-9 has been shown to be associated to tumor burden [18] and levels > 1000 U/ml has been shown to be a negative prognostic factor for survival [19]. In our study, the median survival (12 months) among patients with preoperative CA19-9 level < 200 U/ml was far longer than that among patients with CA19-9 levels > 200 U/ml (8 months).

It has also been reported that high CA19-9 levels are significantly correlated with important histopathologic factors such as major vessel, bile duct, and perineural invasion. These findings indicate that high preoperative CA19-9 levels may predictof histopathologic invasiveness of ICC as well as poor survival [20].

Lymph node status has been shown to be of prognostic significance among patients without distant metastases [4,5]. In a previous study 3- and 5-year survival rates were shown to be 40% and 25% respectively for patients with N0M0 disease compared to 21% and 4% respectively for those with N1M0 disease [21]. In our study, survival was compromised by the presence of lymph node metastasis as demonstrated by both univariate and multivariate analysis. In accordance with our findings lymphatic invasion has been shown to be an independent prognostic factor for survival, and a more important predictor for outcomes of ICC than lymph node metastasis [20].

Hepatolithiasis is a common disease in China, and 5% to 10% of cases of this condition are known to be associated with cholangiocarcinoma [22]. The clinicopathologic features of hepatolithiasis and hepatobiliary cancer (IHHCC) are similar, and the overall survival associated with IHHCC is poor [23]. In our study we found that the presence of intrahepatic duct stones was associated with improved postoperative survival. This observation was contrary to previous findings [5,24] indicating that further research is needed to ascertain the underlying mechanism of action.

In our study, median survival was longer in patients undergoing radical resection (12 months) than in those receiving palliative resection (5 months). However, both survival times were lower than those previously reported (36 and 10 months, respectively) [16]. This may be related to the fact that only 60 (84.5%) of the 81 patients in the study received radical tumor resection.

In common with other studies we found that intrahepatic metastasis was a poor prognostic factor for survival [5,11,16]. Diabetes has also been reported to be a risk factor for the development of ICC [25]. In our study the relationship diabetes and the prognosis of ICC was unclear. However, we did demonstrate that ICC patients with diabetes had a very short survival time.

Prospective validation of our results based on a larger multicenter population is required before our findings can be applied to clinical practice. However, this initial analysis suggests that current or previous HBV infection or vaccination may be associated with significantly better prognosis than in patients without HBV infection. Our findings also highlight the need to improve early diagnostic accuracy in order to allow radical tumor resection in as many patients as possible. Adjuvant chemotherapy appears to prolong survival for patients unable to tolerate radical resection.

## Competing interests

All authors declare that they have no competing interests.

## Authors’ contributions

XL, SS and RL conceived and designed the study. RL and JL drafted the manuscript. RL, SS, XF, JL and LC joined in collecting, analysis and interpretation of data for the work. All authors have read and approved the final manuscript.

## References

[B1] Ikai I Itai Y Okita K Report of the 15th follow-up survey of primary liver cancer Hepatol Res 2004 28 1 21 29 10.1016/j.hepres.2003.08.002 14734147

[B2] Khan SA Thomas HC Davidson BR Cholangiocarcinoma Lancet 2005 366 9493 1303 1310 10.1016/S0140-6736(05)67530-7 16214602

[B3] Wang W-l Guang-yu G Min H Expressionandsignificance of hepatitis Bvirus genes inhumanprimaryintrahepatic cholangiocar-cinomaanditssurroundingtissue Chin J Oncol 1996 18 2 127 129

[B4] Qu ZL Zou SQ Cui NQ Upregulation of human telomerase reverse transcriptase mRNA expression by in vitrotransfection of hepatitis B virus X gene into human hepatocarcinoma and cholangiocarcinoma cells World J Gastroenterol 2005 11 36 5627 5632 16237755 10.3748/wjg.v11.i36.5627 PMC4481478

[B5] Zhang L Cai JQ Zhao JJ Impact of hepatitis B virus infection on outcome following resection for intrahepatic cholangiocarcinoma J Surg Oncol 2010 101 3 233 238 10.1002/jso.21488 20169539

[B6] Zhou HB Wang H Li YQ Hepatitis B virus infection: A favorable prognostic factor for intrahepatic cholangiocarcinoma after resection World J Gastroenterol 2011 17 10 1292 1303 10.3748/wjg.v17.i10.1292 21455328 PMC3068264

[B7] Vitale F Tramuto F Orlando A Can the serological status of anti-HBc alone be considered a sentinel marker for detection of occult HBV infection J Med Virol 2008 80 4 577 582 10.1002/jmv.21121 18297707

[B8] Arase Y Suzuki F Suzuki Y Long-term presence of HBV in the sera of chronic hepatitis B patients with HBsAg seroclearance Intervirology 2007 50 3 161 165 10.1159/000098958 17259734

[B9] Thongprasert S The role of chemotherapy in cholangiocarcinoma Ann Oncol 2005 16 2 ii93 ii96 15958484 10.1093/annonc/mdi712

[B10] Yue M Ying-Chun X Lei T Cytokine-induced killer (CIK) cell therapy for patients with hepatocellular carcinoma: efficacy and safety Exp Hematol & Oncol 2012 1 11 10.1186/2162-3619-1-11 23210562 PMC3514101

[B11] Chen LP Li C Wang C Predictive factors of recurrence for patients with intrahepatic cholangiocarcinoma after hepatectomy Hepatogastroenterology 2012 59 118 1765 1768 22369746 10.5754/hge11820

[B12] Nanashima A Sumida Y Abo T Relationship between pattern of tumor enhancement and clinicopathologic characteristics in intrahepatic cholangiocarcinoma J Surg Oncol 2008 98 7 535 539 10.1002/jso.21142 18814285

[B13] Haruna Y Saito K Spaulding S Identification of bipotential progenitor cells in human liver development Hepatology 1996 23 3 476 481 10.1002/hep.510230312 8617427

[B14] Ding F-x Wang F Yi-ming L Multiepitope peptide-loaded virus-like particles as a vaccine against hepatitis B virus–related hepatocellular carcinoma Hepatology 2009 49 5 1492 1502 10.1002/hep.22816 19206147

[B15] Morise Z Sugioka A Tokoro T Surgery and chemotherapy for intrahepatic cholangiocarcinoma World J Hepatol 2010 2 2 58 64 21160974 10.4254/wjh.v2.i2.58 PMC2998957

[B16] Li F-H Chen X-Q Luo Y-H Prognosis of 84 intrahepatic cholangiocarcinoam patients Chin J Cancer 2009 28 5 528 532 19624884

[B17] Jan YY Yeh CN Yeh TS Prognostic analysis of surgical treatment of periheral cholangiocarcinoma: two decades of experience at chang gung memorial hospital World J Gas-troenterol 2005 11 12 1779 1784 15793863 10.3748/wjg.v11.i12.1779 PMC4305873

[B18] Patel AH Harnois DM Klee GG The utility of CA 19–9 in the diagnoses of cholangiocarcinoma in patients without primary sclerosing cholangitis Am J Gastroenterol 2000 95 1 204 207 10.1111/j.1572-0241.2000.01685.x 10638584

[B19] Ohtsuka M Ito H Kimura F Results of surgical treatment for intrahepatic cholangiocarcinoma and clinicopathological factors influencing survival Br J Surg 2002 89 12 1525 1531 10.1046/j.1365-2168.2002.02268.x 12445060

[B20] Cho SY Park SJ Kim SH Survival analysis of intrahepatic cholangiocarcinoam after resection Ann Surg Oncol 2010 17 7 1823 1830 10.1245/s10434-010-0938-y 20165987

[B21] Nathan H Aloia TA Vauthey J-N A proposed staging system for intrahepatic cholangiocarcinoma Ann Surg Oncol 2009 16 1 14 22 10.1245/s10434-008-0180-z 18987916

[B22] Uenishi T Yamazaki O Yamamoto T Serosal invasion in TNM staging of mass-forming intrahepatic cholangiocarcinoma Hepatobiliary Pancreat Surg 2005 12 6 479 483 10.1007/s00534-005-1026-8 16365823

[B23] Li HY Zhou SJ Li M Diagnosis and cure experience of hepatolithiasis- associated intrahepatic cholangiocarcinoma in 66 patients Asian Pac J Cancer Prev 2012 13 2 725 729 10.7314/APJCP.2012.13.2.725 22524851

[B24] Zhao S-b Cai-de L Dignosis and treatment hepatolithias associated with cholangiocarcinoma:a report of 28 cases Int J Surg 2010 7 37 445 447

[B25] Chun-xing L Zhou J Association between diabetes mellitus and the risk of extrahepatic cholangiocarcinoma: a Meta-analysis Actauniversitats Medicinalis Nedicinalis Nanjing (Natural Science) 2011 6 31 834 837

